# A Convenience

**Published:** 1883-03

**Authors:** 


A Convenience.—Dentists very frequently feel the need of a drop-tube, that may be used for different preparations and medicinal agents. A pair of ordinary foil plyers very nearly fulfil every requirement. They are easily cleaned, are readily inserted into the mouth of a vial, are always at hand when needed, and are certain in their action. If the points be brought in contact they will retain about a minim or drop of any fluid, which may then be conveniently carried into the cavity of a tooth, or into any sinus in the mouth, and when the points are allowed to separate the drop is yielded. A pair of plain plyers should he kept for this special use, and simply wiping them with a piece of chamois skin is quite sufficient to remove most of the topical applications which dentists use.

